# 1-(2-Bromo­meth­yl-1-phenyl­sulfonyl-1*H*-indol-3-yl)propan-1-one

**DOI:** 10.1107/S1600536813031413

**Published:** 2013-11-23

**Authors:** M. Umadevi, V. Saravanan, R. Yamuna, A. K. Mohanakrishnan, G. Chakkaravarthi

**Affiliations:** a Research Scholar (Chemistry), Bharathiyar University, Coimbatore 641 046, Tamilnadu, India; b Department of Organic Chemistry, University of Madras, Guindy Campus, Chennai 600 025, India; c Department of Sciences, Chemistry and Materials Research Lab, Amrita Vishwa Vidyapeetham University, Ettimadai, Coimbatore 641 112, India; d Department of Physics, CPCL Polytechnic College, Chennai 600 068, India

## Abstract

In the title compound, C_18_H_16_BrNO_3_S, the dihedral angle between the phenyl ring and the indole ring system is 89.91 (11)°. The mol­ecular structure features weak C—H⋯O and C—H⋯Br hydrogen bonds. In the crystal, mol­ecules are linked by weak C—H⋯O hydrogen bonds, forming chains along the *a-*axis direction. The chains are further linked by C—H⋯π inter­actions, forming a layer parallel to the *ab* plane.

## Related literature

For the biological activity of indole derivatives, see: Chai *et al.* (2006 ▶); Nieto *et al.* (2005 ▶). For related structures, see: Chakkaravarthi *et al.* (2008 ▶, 2010 ▶). For details of the configuration at the S atom, see: Bassindale (1984 ▶). For details of N-atom hybridization, see: Beddoes *et al.* (1986 ▶).
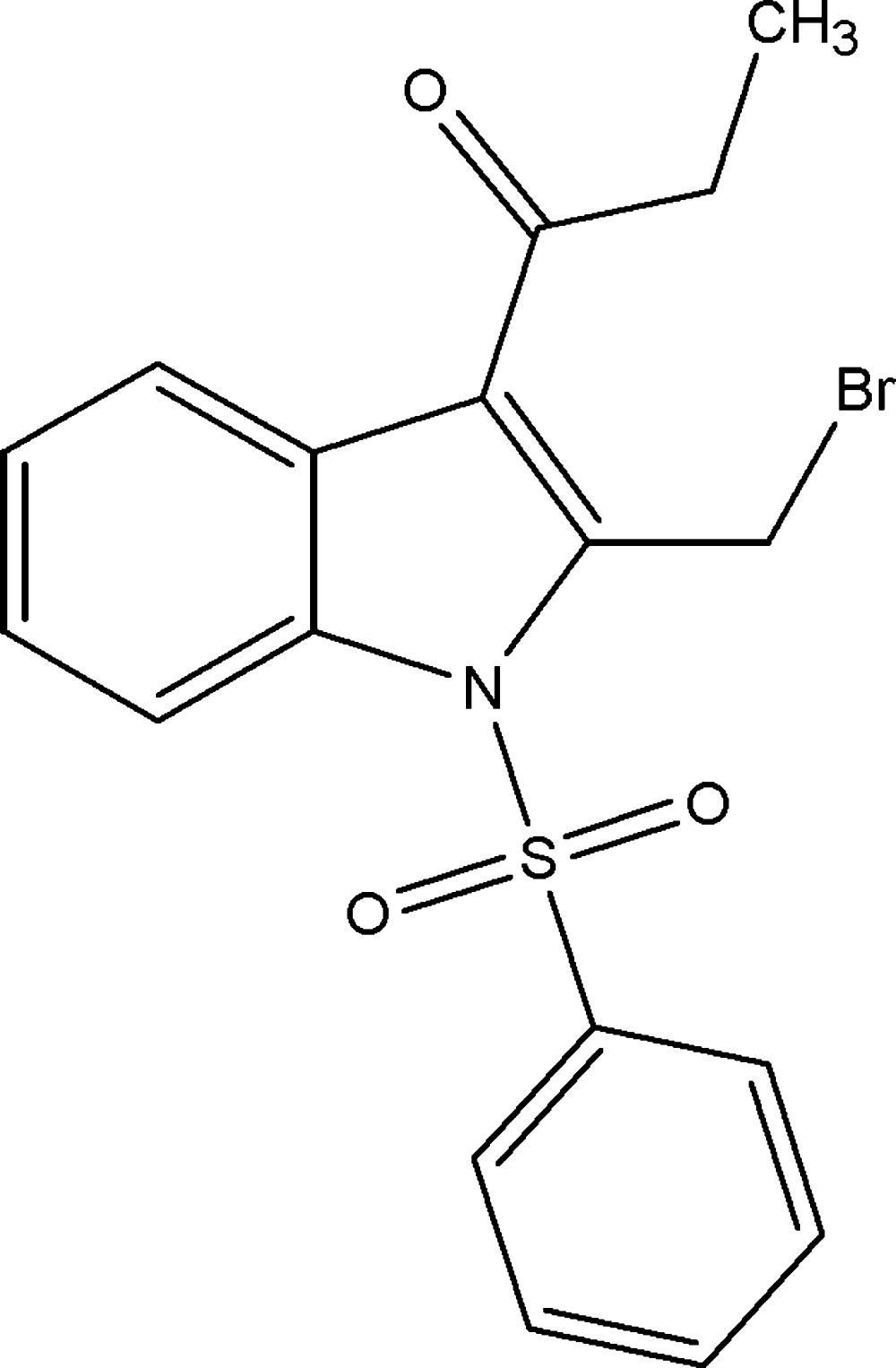


## Experimental

### 

#### Crystal data

C_18_H_16_BrNO_3_S
*M* *_r_* = 406.29
Monoclinic, 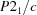

*a* = 10.2772 (7) Å
*b* = 8.6610 (6) Å
*c* = 18.8980 (14) Å
β = 90.676 (2)°
*V* = 1682.0 (2) Å^3^
*Z* = 4
Mo *K*α radiation
μ = 2.58 mm^−1^
*T* = 295 K
0.38 × 0.34 × 0.30 mm

#### Data collection

Bruker Kappa APEXII diffractometer
Absorption correction: multi-scan (*SADABS*; Sheldrick, 1996 ▶) *T*_min_ = 0.397, *T*_max_ = 0.461
25944 measured reflections
7303 independent reflections
3726 reflections with *I* > 2σ(*I*)
*R*_int_ = 0.036

#### Refinement

*R*[*F* ^2^ > 2σ(*F* ^2^)] = 0.052
*wR*(*F* ^2^) = 0.133
*S* = 1.00
7303 reflections
218 parameters
H-atom parameters constrained
Δρ_max_ = 1.11 e Å^−3^
Δρ_min_ = −0.90 e Å^−3^

### 

Data collection: *APEX2* (Bruker, 2004 ▶); cell refinement: *SAINT* (Bruker, 2004 ▶); data reduction: *SAINT*; program(s) used to solve structure: *SHELXS97* (Sheldrick, 2008 ▶); program(s) used to refine structure: *SHELXL97* (Sheldrick, 2008 ▶); molecular graphics: *PLATON* (Spek, 2009 ▶); software used to prepare material for publication: *SHELXL97*.

## Supplementary Material

Crystal structure: contains datablock(s) I, global. DOI: 10.1107/S1600536813031413/is5323sup1.cif

Structure factors: contains datablock(s) I. DOI: 10.1107/S1600536813031413/is5323Isup2.hkl

Supplementary material file. DOI: 10.1107/S1600536813031413/is5323Isup3.cml

Additional supplementary materials:  crystallographic information; 3D view; checkCIF report

## Figures and Tables

**Table 1:** Hydrogen-bond geometry (Å, °) *Cg*2 and *Cg*3 are the centroids of the C1–C6 and C9–C14 rings, respectively.

*D*—H⋯*A*	*D*—H	H⋯*A*	*D*⋯*A*	*D*—H⋯*A*
C6—H6⋯Br1	0.93	2.89	3.815 (2)	171
C13—H13⋯O2	0.93	2.39	2.978 (3)	121
C15—H15*B*⋯O1	0.97	2.15	2.833 (3)	126
C2—H2⋯O3^i^	0.93	2.59	3.191 (3)	123
C15—H15*A*⋯*Cg*3^ii^	0.97	2.74	3.486 (3)	134
C18—H18*B*⋯*Cg*2^iii^	0.96	2.66	3.616 (3)	174

Symmetry codes: (i) 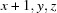; (ii) 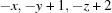; (iii) 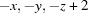.

**Fig. 1.**
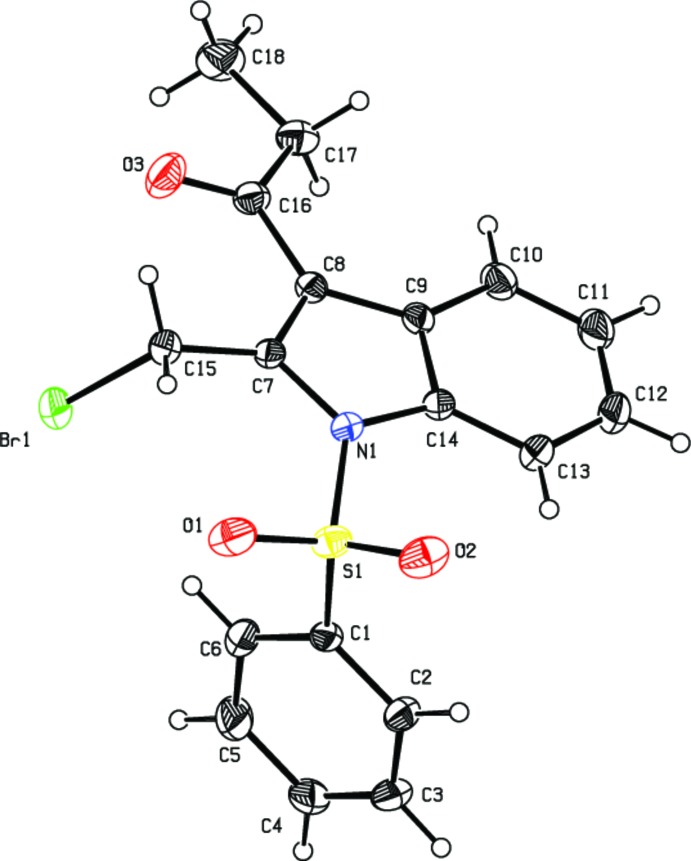
The molecular structure of the title compound, with atom labels and 30% probability displacement ellipsoids for non-H atoms.

**Fig. 2.**
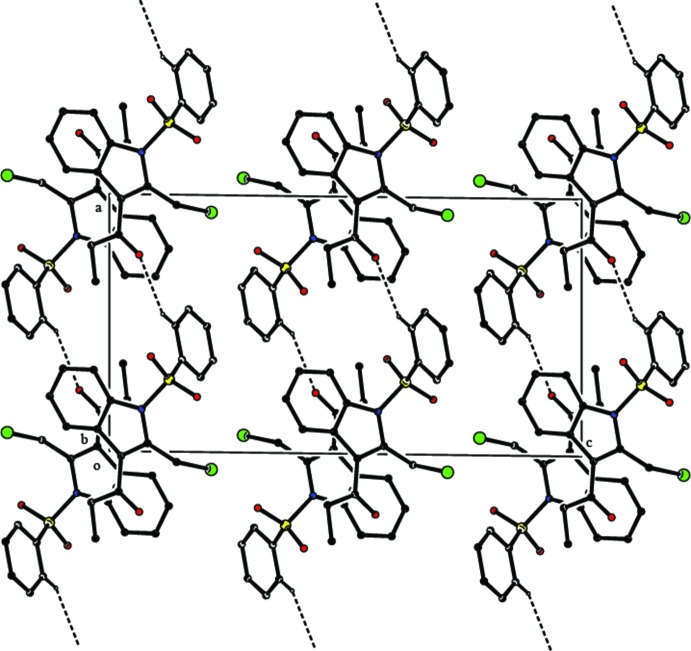
A packing diagram of the title compound viewed down the b axis. Intermolecular hydrogen bonds are shown as dashed lines. H atoms not involving hydrogen bonding have been omitted.

## supplementary crystallographic information

### 1. Comment

Indole derivatives exhibit antihepatitis B virus (Chai *et al.*, 2006) and antibacterial (Nieto *et al.*, 2005) activities. We herein report the crystal structure of the title compound (I) (Fig. 1). The bond distances of (I) are comparable with the reported similar structures (Chakkaravarthi *et al.*, 2008, 2010). The bond angles around atom S1 show significant deviation from ideal tetrahedral value [O1—S1—O2 = 120.16 (13)° and N1—S1—C1 = 105.11 (10)°] due to Thorpe-Ignold effect (Bassindale, 1984). The sum of the bond angles around N1 (358.05°) indicates the *sp*^2^ hybridization of N1 atom (Beddoes *et al.*, 1986).

The indole ring system is planar, with the dihedral angle between the two rings (N1/C7/C8/C9/C14) and (C9–C14) is 2.00 (12)°. The phenyl ring (C1–C6) makes the dihedral angle of 89.91 (11)° with the indole ring system. The molecular structure is stabilized by weak intramolecular C—H···O and C—H···Br hydrogen bonds (Table 1). The crystal structure exhibit weak intermolecular C—H···O and C—H···π (Table 1 & Fig. 2) interactions.

### 2. Experimental

A mixture of 1-[2-methyl-1-(phenylsulfonyl)-1*H*-indol-3-yl]propan-1-one (15 g, 45.87 mmol) and *N*-bromosuccinimide (9.8 g, 55 mmol) in dry CCl_4_ (250 ml) containing a catalytic amount of 2,2'-azobis(isobutyronitrile) (50 mg) was refluxed for 3 h. Then, the reaction mixture was cooled to room temperature, filtered off the floated succinimide through Na_2_SO_4_ pad and washed with CCl_4_ (20 ml). Removal of the solvent followed by trituration of the crude product with MeOH (50 ml) gave the title compound, suitable for X-ray diffraction quality.

### 3. Refinement

H atoms were positioned geometrically and refined using a riding model with C—H = 0.93–0.97 Å, and with *U*_iso_(H) = 1.2*U*_eq_(C) or 1.5*U*_eq_(C_methyl_).

### Crystal data

**Table:**

C_18_H_16_BrNO_3_S	*F*(000) = 824
*M**_r_* = 406.29	*D*_x_ = 1.604 Mg m^−^^3^
Monoclinic, *P*2_1_/*c*	Mo *K*α radiation, λ = 0.71073 Å
Hall symbol: -P 2ybc	Cell parameters from 5675 reflections
*a* = 10.2772 (7) Å	θ = 2.6–28.2°
*b* = 8.6610 (6) Å	µ = 2.58 mm^−^^1^
*c* = 18.8980 (14) Å	*T* = 295 K
β = 90.676 (2)°	Block, colourless
*V* = 1682.0 (2) Å^3^	0.38 × 0.34 × 0.30 mm
*Z* = 4	

### Data collection

**Table:**

Bruker Kappa APEXII diffractometer	7303 independent reflections
Radiation source: fine-focus sealed tube	3726 reflections with *I* > 2σ(*I*)
Graphite monochromator	*R*_int_ = 0.036
ω and φ scan	θ_max_ = 35.0°, θ_min_ = 2.2°
Absorption correction: multi-scan (*SADABS*; Sheldrick, 1996)	*h* = −16→15
*T*_min_ = 0.397, *T*_max_ = 0.461	*k* = −13→13
25944 measured reflections	*l* = −30→30

### Refinement

**Table:**

Refinement on *F*^2^	Primary atom site location: structure-invariant direct methods
Least-squares matrix: full	Secondary atom site location: difference Fourier map
*R*[*F*^2^ > 2σ(*F*^2^)] = 0.052	Hydrogen site location: inferred from neighbouring sites
*wR*(*F*^2^) = 0.133	H-atom parameters constrained
*S* = 1.00	*w* = 1/[σ^2^(*F*_o_^2^) + (0.0528*P*)^2^ + 0.8633*P*] where *P* = (*F*_o_^2^ + 2*F*_c_^2^)/3
7303 reflections	(Δ/σ)_max_ < 0.001
218 parameters	Δρ_max_ = 1.11 e Å^−^^3^
0 restraints	Δρ_min_ = −0.90 e Å^−^^3^

### Special details

**Table:**

Geometry. All e.s.d.'s (except the e.s.d. in the dihedral angle between two l.s. planes) are estimated using the full covariance matrix. The cell e.s.d.'s are taken into account individually in the estimation of e.s.d.'s in distances, angles and torsion angles; correlations between e.s.d.'s in cell parameters are only used when they are defined by crystal symmetry. An approximate (isotropic) treatment of cell e.s.d.'s is used for estimating e.s.d.'s involving l.s. planes.

### Fractional atomic coordinates and isotropic or equivalent isotropic displacement parameters (Å^2^)

**Table:**

	*x*	*y*	*z*	*U*_iso_*/*U*_eq_	
C1	0.3590 (2)	0.2855 (3)	1.15387 (11)	0.0316 (4)	
C2	0.4906 (2)	0.2669 (3)	1.14030 (14)	0.0388 (5)	
H2	0.5349	0.3387	1.1130	0.047*	
C3	0.5545 (2)	0.1404 (3)	1.16792 (14)	0.0478 (6)	
H3	0.6429	0.1276	1.1599	0.057*	
C4	0.4891 (3)	0.0341 (4)	1.20682 (14)	0.0527 (7)	
H4	0.5334	−0.0508	1.2251	0.063*	
C5	0.3573 (3)	0.0506 (4)	1.21952 (16)	0.0594 (8)	
H5	0.3134	−0.0236	1.2456	0.071*	
C6	0.2917 (2)	0.1775 (3)	1.19338 (14)	0.0468 (6)	
H6	0.2035	0.1906	1.2021	0.056*	
C7	0.03158 (18)	0.3688 (2)	1.08099 (11)	0.0276 (4)	
C8	−0.0180 (2)	0.2867 (2)	1.02518 (11)	0.0290 (4)	
C9	0.0851 (2)	0.2600 (2)	0.97521 (11)	0.0304 (4)	
C10	0.0935 (2)	0.1861 (3)	0.90938 (12)	0.0407 (5)	
H10	0.0217	0.1359	0.8898	0.049*	
C11	0.2110 (3)	0.1894 (3)	0.87400 (14)	0.0492 (7)	
H11	0.2173	0.1407	0.8303	0.059*	
C12	0.3188 (3)	0.2635 (3)	0.90221 (14)	0.0491 (7)	
H12	0.3957	0.2646	0.8768	0.059*	
C13	0.3152 (2)	0.3356 (3)	0.96705 (13)	0.0411 (6)	
H13	0.3880	0.3847	0.9861	0.049*	
C14	0.19702 (19)	0.3317 (3)	1.00296 (11)	0.0306 (4)	
C15	−0.0399 (2)	0.4324 (3)	1.14247 (12)	0.0358 (5)	
H15A	−0.1235	0.4717	1.1264	0.043*	
H15B	0.0090	0.5179	1.1625	0.043*	
C16	−0.1576 (2)	0.2394 (3)	1.02006 (13)	0.0355 (5)	
C17	−0.2006 (2)	0.1315 (3)	0.96217 (14)	0.0419 (6)	
H17A	−0.1440	0.0418	0.9621	0.050*	
H17B	−0.1922	0.1829	0.9169	0.050*	
C18	−0.3408 (3)	0.0787 (4)	0.97091 (16)	0.0561 (7)	
H18A	−0.3494	0.0268	1.0155	0.084*	
H18B	−0.3639	0.0093	0.9332	0.084*	
H18C	−0.3975	0.1668	0.9696	0.084*	
N1	0.16494 (16)	0.3982 (2)	1.06867 (9)	0.0299 (4)	
O1	0.21453 (16)	0.5195 (2)	1.18571 (10)	0.0523 (5)	
O2	0.36972 (17)	0.5470 (2)	1.08811 (11)	0.0523 (5)	
O3	−0.23528 (17)	0.2863 (3)	1.06323 (11)	0.0571 (5)	
S1	0.28085 (5)	0.45547 (7)	1.12687 (3)	0.03548 (14)	
Br1	−0.06733 (3)	0.27582 (4)	1.215850 (15)	0.06069 (12)	

### Atomic displacement parameters (Å^2^)

**Table:**

	*U*^11^	*U*^22^	*U*^33^	*U*^12^	*U*^13^	*U*^23^
C1	0.0272 (9)	0.0409 (12)	0.0268 (10)	0.0035 (9)	−0.0028 (8)	−0.0044 (9)
C2	0.0273 (10)	0.0475 (14)	0.0417 (13)	−0.0015 (9)	0.0007 (9)	−0.0020 (11)
C3	0.0305 (11)	0.0644 (18)	0.0484 (15)	0.0119 (11)	−0.0046 (10)	−0.0013 (13)
C4	0.0490 (14)	0.0675 (19)	0.0415 (14)	0.0214 (14)	−0.0025 (12)	0.0139 (13)
C5	0.0545 (16)	0.072 (2)	0.0515 (16)	0.0105 (15)	0.0129 (13)	0.0290 (15)
C6	0.0332 (11)	0.0619 (17)	0.0456 (15)	0.0068 (11)	0.0090 (10)	0.0126 (13)
C7	0.0253 (8)	0.0293 (11)	0.0281 (10)	0.0034 (8)	0.0001 (8)	0.0040 (8)
C8	0.0285 (9)	0.0303 (11)	0.0281 (10)	0.0006 (8)	−0.0007 (8)	0.0036 (8)
C9	0.0324 (10)	0.0318 (11)	0.0270 (10)	0.0045 (8)	0.0001 (8)	0.0047 (8)
C10	0.0456 (13)	0.0457 (14)	0.0307 (12)	0.0055 (11)	−0.0012 (10)	−0.0033 (10)
C11	0.0534 (15)	0.0605 (17)	0.0339 (13)	0.0191 (13)	0.0064 (11)	−0.0038 (12)
C12	0.0409 (12)	0.0657 (18)	0.0411 (14)	0.0144 (12)	0.0138 (11)	0.0046 (13)
C13	0.0302 (10)	0.0525 (15)	0.0409 (13)	0.0053 (10)	0.0052 (10)	0.0047 (11)
C14	0.0283 (9)	0.0341 (11)	0.0294 (10)	0.0056 (8)	0.0008 (8)	0.0040 (9)
C15	0.0331 (10)	0.0424 (13)	0.0321 (11)	0.0050 (9)	0.0036 (9)	−0.0016 (10)
C16	0.0308 (10)	0.0373 (13)	0.0383 (12)	−0.0023 (9)	−0.0034 (9)	0.0044 (10)
C17	0.0404 (12)	0.0399 (14)	0.0452 (14)	−0.0030 (10)	−0.0091 (11)	0.0015 (11)
C18	0.0482 (15)	0.0594 (18)	0.0606 (18)	−0.0179 (13)	−0.0115 (13)	−0.0020 (15)
N1	0.0244 (7)	0.0356 (10)	0.0296 (9)	0.0016 (7)	−0.0013 (7)	−0.0002 (8)
O1	0.0429 (9)	0.0590 (12)	0.0548 (11)	0.0097 (8)	−0.0095 (8)	−0.0286 (9)
O2	0.0412 (9)	0.0383 (10)	0.0774 (14)	−0.0094 (8)	−0.0049 (9)	0.0062 (10)
O3	0.0341 (9)	0.0783 (14)	0.0593 (13)	−0.0117 (9)	0.0086 (8)	−0.0156 (11)
S1	0.0297 (2)	0.0333 (3)	0.0434 (3)	0.0002 (2)	−0.0058 (2)	−0.0068 (2)
Br1	0.04826 (16)	0.0928 (3)	0.04125 (16)	0.01267 (15)	0.01231 (12)	0.02479 (15)

### Geometric parameters (Å, º)

**Table:**

C1—C6	1.386 (3)	C11—C12	1.382 (4)
C1—C2	1.389 (3)	C11—H11	0.9300
C1—S1	1.750 (2)	C12—C13	1.376 (4)
C2—C3	1.376 (4)	C12—H12	0.9300
C2—H2	0.9300	C13—C14	1.399 (3)
C3—C4	1.361 (4)	C13—H13	0.9300
C3—H3	0.9300	C14—N1	1.411 (3)
C4—C5	1.385 (4)	C15—Br1	1.963 (2)
C4—H4	0.9300	C15—H15A	0.9700
C5—C6	1.378 (4)	C15—H15B	0.9700
C5—H5	0.9300	C16—O3	1.218 (3)
C6—H6	0.9300	C16—C17	1.502 (3)
C7—C8	1.366 (3)	C17—C18	1.522 (3)
C7—N1	1.416 (2)	C17—H17A	0.9700
C7—C15	1.488 (3)	C17—H17B	0.9700
C8—C9	1.446 (3)	C18—H18A	0.9600
C8—C16	1.494 (3)	C18—H18B	0.9600
C9—C10	1.403 (3)	C18—H18C	0.9600
C9—C14	1.403 (3)	N1—S1	1.6864 (18)
C10—C11	1.387 (4)	O1—S1	1.4236 (18)
C10—H10	0.9300	O2—S1	1.4193 (19)
			
C6—C1—C2	121.0 (2)	C12—C13—C14	117.0 (2)
C6—C1—S1	119.62 (17)	C12—C13—H13	121.5
C2—C1—S1	119.21 (19)	C14—C13—H13	121.5
C3—C2—C1	118.9 (2)	C13—C14—C9	122.8 (2)
C3—C2—H2	120.6	C13—C14—N1	129.1 (2)
C1—C2—H2	120.6	C9—C14—N1	108.08 (17)
C4—C3—C2	120.5 (2)	C7—C15—Br1	111.86 (16)
C4—C3—H3	119.8	C7—C15—H15A	109.2
C2—C3—H3	119.8	Br1—C15—H15A	109.2
C3—C4—C5	120.9 (2)	C7—C15—H15B	109.2
C3—C4—H4	119.5	Br1—C15—H15B	109.2
C5—C4—H4	119.5	H15A—C15—H15B	107.9
C6—C5—C4	119.7 (3)	O3—C16—C8	120.1 (2)
C6—C5—H5	120.2	O3—C16—C17	120.4 (2)
C4—C5—H5	120.2	C8—C16—C17	119.5 (2)
C5—C6—C1	119.1 (2)	C16—C17—C18	112.2 (2)
C5—C6—H6	120.5	C16—C17—H17A	109.2
C1—C6—H6	120.5	C18—C17—H17A	109.2
C8—C7—N1	108.64 (18)	C16—C17—H17B	109.2
C8—C7—C15	127.79 (19)	C18—C17—H17B	109.2
N1—C7—C15	123.36 (19)	H17A—C17—H17B	107.9
C7—C8—C9	108.54 (18)	C17—C18—H18A	109.5
C7—C8—C16	122.81 (19)	C17—C18—H18B	109.5
C9—C8—C16	128.6 (2)	H18A—C18—H18B	109.5
C10—C9—C14	118.3 (2)	C17—C18—H18C	109.5
C10—C9—C8	134.9 (2)	H18A—C18—H18C	109.5
C14—C9—C8	106.79 (19)	H18B—C18—H18C	109.5
C11—C10—C9	118.8 (2)	C14—N1—C7	107.94 (17)
C11—C10—H10	120.6	C14—N1—S1	121.55 (14)
C9—C10—H10	120.6	C7—N1—S1	128.56 (14)
C12—C11—C10	121.5 (2)	O2—S1—O1	120.16 (13)
C12—C11—H11	119.3	O2—S1—N1	106.36 (11)
C10—C11—H11	119.3	O1—S1—N1	106.44 (9)
C13—C12—C11	121.6 (2)	O2—S1—C1	108.93 (11)
C13—C12—H12	119.2	O1—S1—C1	108.79 (11)
C11—C12—H12	119.2	N1—S1—C1	105.11 (10)
			
C6—C1—C2—C3	−1.2 (4)	N1—C7—C15—Br1	−103.6 (2)
S1—C1—C2—C3	173.4 (2)	C7—C8—C16—O3	7.0 (4)
C1—C2—C3—C4	1.2 (4)	C9—C8—C16—O3	−171.7 (2)
C2—C3—C4—C5	−0.1 (5)	C7—C8—C16—C17	−171.8 (2)
C3—C4—C5—C6	−1.0 (5)	C9—C8—C16—C17	9.5 (3)
C4—C5—C6—C1	0.9 (5)	O3—C16—C17—C18	−6.7 (4)
C2—C1—C6—C5	0.2 (4)	C8—C16—C17—C18	172.2 (2)
S1—C1—C6—C5	−174.4 (2)	C13—C14—N1—C7	177.2 (2)
N1—C7—C8—C9	0.5 (2)	C9—C14—N1—C7	−0.8 (2)
C15—C7—C8—C9	175.3 (2)	C13—C14—N1—S1	−17.4 (3)
N1—C7—C8—C16	−178.40 (19)	C9—C14—N1—S1	164.61 (15)
C15—C7—C8—C16	−3.7 (3)	C8—C7—N1—C14	0.2 (2)
C7—C8—C9—C10	−179.2 (2)	C15—C7—N1—C14	−174.9 (2)
C16—C8—C9—C10	−0.4 (4)	C8—C7—N1—S1	−163.90 (16)
C7—C8—C9—C14	−1.0 (2)	C15—C7—N1—S1	21.1 (3)
C16—C8—C9—C14	177.8 (2)	C14—N1—S1—O2	49.4 (2)
C14—C9—C10—C11	−1.1 (3)	C7—N1—S1—O2	−148.47 (19)
C8—C9—C10—C11	176.9 (2)	C14—N1—S1—O1	178.65 (18)
C9—C10—C11—C12	0.0 (4)	C7—N1—S1—O1	−19.2 (2)
C10—C11—C12—C13	0.8 (4)	C14—N1—S1—C1	−66.04 (19)
C11—C12—C13—C14	−0.5 (4)	C7—N1—S1—C1	96.1 (2)
C12—C13—C14—C9	−0.7 (4)	C6—C1—S1—O2	179.4 (2)
C12—C13—C14—N1	−178.4 (2)	C2—C1—S1—O2	4.7 (2)
C10—C9—C14—C13	1.5 (3)	C6—C1—S1—O1	46.8 (2)
C8—C9—C14—C13	−177.0 (2)	C2—C1—S1—O1	−127.98 (19)
C10—C9—C14—N1	179.7 (2)	C6—C1—S1—N1	−66.9 (2)
C8—C9—C14—N1	1.1 (2)	C2—C1—S1—N1	118.35 (19)
C8—C7—C15—Br1	82.3 (2)		

### Hydrogen-bond geometry (Å, º)

*Cg*2 and *Cg*3 are the centroids of the C1–C6 and C9–C14 rings, respectively.

**Table:**

*D*—H···*A*	*D*—H	H···*A*	*D*···*A*	*D*—H···*A*
C6—H6···Br1	0.93	2.89	3.815 (2)	171
C13—H13···O2	0.93	2.39	2.978 (3)	121
C15—H15*B*···O1	0.97	2.15	2.833 (3)	126
C2—H2···O3^i^	0.93	2.59	3.191 (3)	123
C15—H15*A*···*Cg*3^ii^	0.97	2.74	3.486 (3)	134
C18—H18*B*···*Cg*2^iii^	0.96	2.66	3.616 (3)	174

Symmetry codes: (i) *x*+1, *y*, *z*; (ii) −*x*, −*y*+1, −*z*+2; (iii) −*x*, −*y*, −*z*+2.
